# Material-driven glucose responsiveness in insulin delivery systems: carrier selection and performance

**DOI:** 10.3389/fendo.2026.1825247

**Published:** 2026-05-08

**Authors:** Zhihan Li, Jingge Jiang, Ziyu Zhu, Cheng Luo, Chunyi Xu, Chuanpin Chen, Guoqin Ren, Ruofei Huang, Dongjuan He

**Affiliations:** 1 Department of Pharmacy, Xiangya School of Pharmaceutical Sciences, Central South University, Changsha, China; 2 The First Affiliated Hospital of Hunan Normal University/Hunan Provincial People’s Hospital, Changsha, China; 3 Quzhou People’s Hospital Affiliated of Wenzhou Medical University, Quzhou, China; 4 Zhejiang University, Zhejiang, China; 5 Hunan Institute of Drug Control, Changsha, China; 6 Yongkang First People’s Hospital of Wenzhou Medical University, Yongkang, China; 7 The Second People’s Hospital of Quzhou, Quzhou, China

**Keywords:** delivery systems, diabetes mellitus, glucose responsiveness, insulin, the carrier materials

## Abstract

Conventional subcutaneous insulin therapy faces limitations in patient adherence and carries a persistent risk of hypoglycemia, primarily due to the challenges of achieving precise dosing. To address this, glucose-responsive insulin delivery systems have been developed to autonomously adjust insulin release in direct response to real-time fluctuations in blood glucose levels. This approach offers a promising pathway toward achieving more physiological glycemic control. This review systematically summarizes recent advances in the design of such intelligent systems, with a focus on two fundamentally interconnected components: the ongoing evolution of glucose-sensing mechanisms, which encompass enzymatic, non-enzymatic, and transporter-mediated strategies, and the parallel development of advanced carrier materials, including metal-organic frameworks, smart polymers, and bio-derived materials. In addition to outlining these technological developments, this work provides a balanced analysis of the operational principles, relative merits, and key translational challenges inherent in current system designs, with particular attention to long-term biocompatibility, stability, and safety. By presenting a coherent synthesis of the field’s current landscape and its potential future directions, this review aims to inform and support the rational development of next-generation glucose-responsive insulin therapies with stronger prospects for clinical translation.

## 1 Introduction

Diabetes Mellitus (1–4) is a metabolic disease characterized by hyperglycemia and it is classified as type 1 or type 2 diabetes (5, 6) based on its etiology. However, in type 1 diabetes and advanced type 2 diabetes, conventional oral hypoglycemic agents are often ineffective or insufficient, making exogenous insulin replacement the cornerstone of therapy (7, 8). Although immunotherapy (9, 10) and stem cell therapy transformative potential for restoring endogenous insulin secretion, their widespread clinical translation remains constrained by formidable challenges, including systemic safety concerns, exorbitant procedural costs, and limited donor availability. Consequently, insulin replacement therapy continues to be the primary intervention for these patients. However, one of the major challenges associated with insulin replacement therapy is the difficulty in achieving precise insulin dosage control, which can lead to hypoglycemia, coma, and even death. In addition, pain associated with frequent subcutaneous injections results in poor patient compliance (11, 12). The concept of a closed-loop insulin delivery system was first introduced by Kadish in the early 1960s (13). In recent years, this concept has been significantly developed and translated into clinical practice (14–17). This system aims to integrate continuous glucose monitoring with real-time insulin infusion, enabling automated dosage adjustment (18–22). Several glucose-responsive insulin devices (23, 24) have been designed for long-term glucose monitoring and precise insulin administration, as exemplified by advances in phenylboronic acid-based hydrogels, glucose oxidase-encapsulating metal–organic frameworks, and transdermal microneedle patches (25–31).

Although researchers have explored various ways to deliver insulin, many of these alternatives face significant limitations related to bioavailability, primarily due to challenges such as the instability of insulin at high temperatures and the poor permeability of biological barriers (32). In addition, the broader implementation of pump devices for continuous subcutaneous insulin infusion faces several impediments, including their prohibitive cost, the latent risk of skin infection, and limited portability. These challenges have catalyzed a concerted effort among researchers to innovate and develop intelligent insulin delivery systems that function within the body itself (33–38). These advanced systems are typically composed of two integral components: a glucose reaction unit and an insulin carrier. The glucose reaction unit operates as a highly sensitive sensor, capable of detecting minute fluctuations in blood glucose levels with remarkable precision. Concurrently, the insulin carrier is engineered with tunable properties including hydrophilicity, crosslinking density, and pH sensitivity (39). These properties govern the insulin release rate in response to ambient glucose levels. The development of such systems represents a paradigm shift in diabetes management, offering a more responsive, patient-friendly, and cost-effective alternative to traditional insulin delivery methods (40).

**Figure 1:**
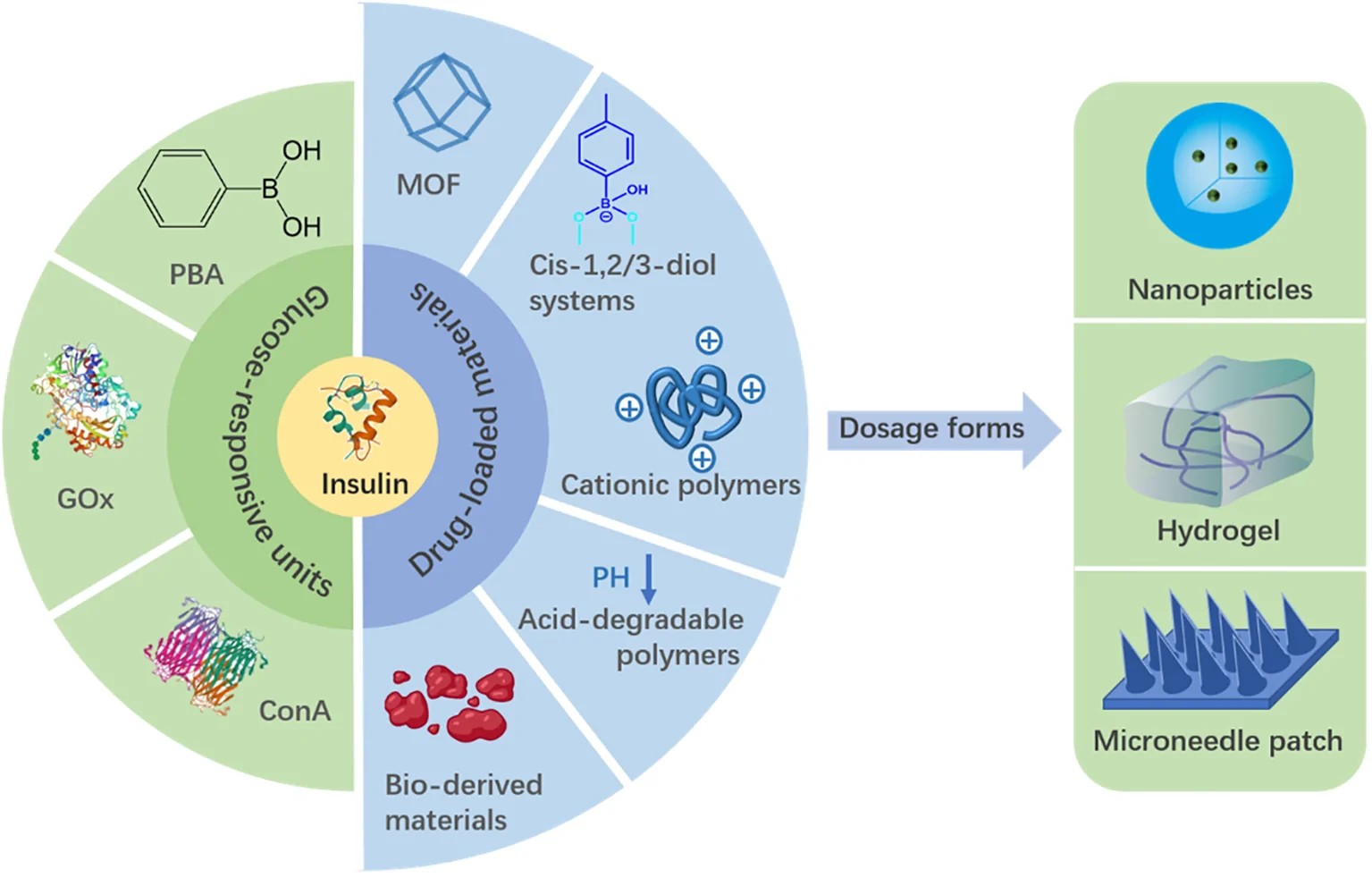
General diagram of glucose-responsive units, drug-loaded materials, and main dosage forms. This schematic illustrates the integrated design of intelligent insulin delivery systems where glucose-responsive units function as molecular sensors to detect glycemic fluctuations and initiate corresponding structural changes within the drug-loaded materials. These smart platforms are further formulated into nanoparticles, hydrogels, or microneedle patches to enable precise and autonomous closed-loop blood glucose regulation.

In the design of glucose-responsive insulin delivery systems, the selection of suitable carrier materials is crucial for achieving a rapid glycemic response, slow-release properties, and high biocompatibility. This review aims to summarize recent advances in glucose-responsive long-acting insulin delivery. We begin by introducing commonly used glucose-responsive units, and subsequently delves into the classification, function, and selection of various carrier materials (**Figure 1**). We anticipate that this review will aid researchers in comparing different technological options and guide the rational design of next-generation smart insulin delivery systems, ultimately aiming to improve clinical outcomes.

## 2 Glucose-responsive units

**Figure 2:**
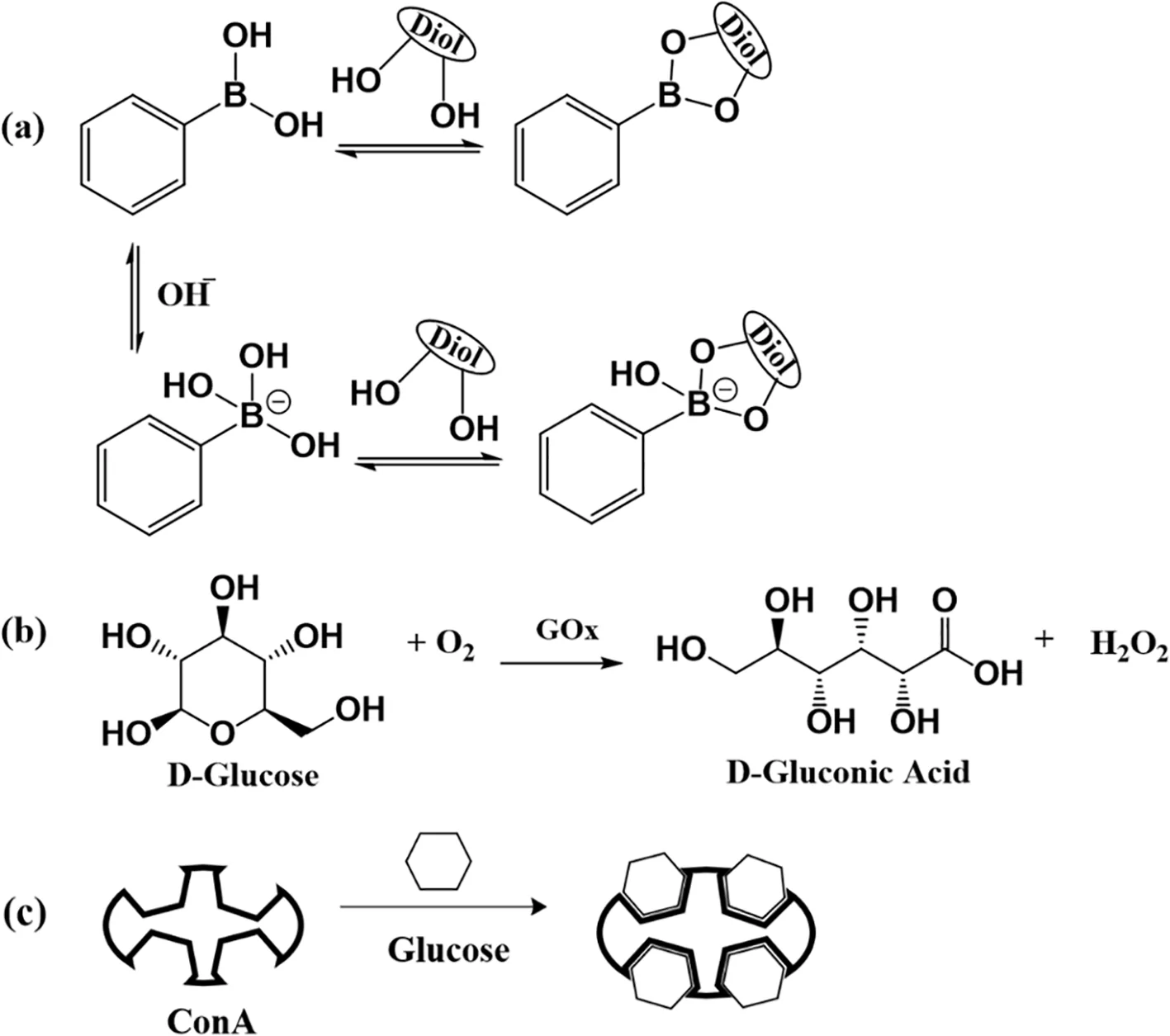
**(a)** Equilibrium between charged and uncharged PBA and its esters. **(b)** Catalytic principle of GOx: D-glucose is converted to D-gluconic acid and H_2_O_2_ catalyzed by GOx. **(c)** ConA has four sites that can be linked to glucose.

Glucose-responsive units serve as the core of intelligent delivery systems, triggering cascade responses through specific interactions with glucose. Phenylboronic acid (PBA) (41), glucose oxidase (GOx) (42), and concanavalin A (ConA) (43) are the most commonly used glucose response units at present. As shown in **Figure 2**, PBA is a Lewis acid containing a phenyl substituent and two hydroxyl groups attached to boron, which bind OH^-^ in solution to form a stable formation of tetrahydroxy-homo boronic acid [B (OH)_4_^-^] complexes; the latter with cis-1,2/3-dihydroxyl sugars are more strongly bound. As the most widely used chemical sensing molecule, the key advantages of PBA lie in its non-enzymatic nature and excellent stability. GOx catalyzes the conversion of glucose to gluconic acid and H_2_O_2_. ConA is a lectin that binds glucose, and ConA has four glucose-binding sites. This multivalent nature allows ConA to function as a reversible cross-linking agent in the construction of glucose-sensitive matrices, where it can undergo competitive binding between endogenous glucose and synthetic glucans or glucose-modified polymers. For instance, Li et al. designed a novel glucose biosensor containing ConA and glucan utilizing the competitive binding mechanism between ConA and glucan. In this intelligent sensing system, ConA serves as a biological cross linker that binds with glucan molecules to form a stable and interconnected molecular network. When exposed to an environment with fluctuating glucose levels, the free glucose molecules compete for the binding sites on ConA and displace the glucan chains due to their dynamic affinity. This competitive displacement triggers a concentration dependent dissociation of the cross linked framework, resulting in detectable changes in the physical properties of the system to enable precise monitoring of glucose concentrations (44).

PBA and its congeners have garnered considerable acclaim as glucose-responsive entities, owing to their innate capacity to engage with the cis-diol configuration of glucose, culminating in the formation of cyclic boronic esters (45, 46). Recent scientific literature demonstrates increasing focus on the integration of PBAs (47–51) into various polymeric matrices to develop intelligent insulin delivery systems. These modified PBA systems are ingeniously crafted to lower the pKa of PBA, thereby augmenting its sensitivity and responsiveness to glucose levels within the physiological milieu (48, 52, 53).

To improve the glucose responsiveness of PBA, various diboronate materials have been developed (54). For instance, Xiang et al. (55) designed materials that can simultaneously bind two sites on a glucose molecule, improving glucose sensitivity and enabling a rapid response. These materials are also less influenced by non-glucose diol compounds, thereby significantly improving the specificity for glucose molecules. Furthermore, glucose-responsive sensors using PBA hydrogel systems have attracted the attention of researchers (56). In addition to this, H. J. Sim and co-workers have developed a self-helical hydrogel fiber based on PBA for artificial muscles, glucose sensors, and drug delivery (57). H. Guo et al. have developed a glucose-responsive ionic skin based on PBA (58). Another noteworthy innovation involves the design of a hydrogel system based on PBA and GOx to create a dual glucose response mechanism, enabling precise control over insulin release within the hydrogel (59).

GOx remains a fundamental component in enzymatic delivery systems due to its ability to catalyze the conversion of glucose to gluconate and hydrogen peroxide, facilitating multiple responsive actions, including pH, H_2_O_2_, and glucose responsiveness. It plays an important role in insulin delivery (60), monitoring of blood glucose (61, 62) and in the delivery of other drugs that require an acidic environment for release, such as anticancer drugs (63). The pH responsiveness of GOx has been investigated in recent years (64–66). It has also been used as a prophylactic pathway for glucagon delivery, in combination with a glucose stability material, against subsequent hypoglycemic episodes (67).

In a study conducted by Xu et al., a glucose-responsive nanosystem was designed as a delivery vehicle for oral insulin administration (68). The system employed konjac glucomannan cross-linked with ConA to co-encapsulate insulin (ConA-INS-KGM). This system demonstrated stability in the gastrointestinal tract, and glucose competed with ConA for binding during hyperglycemic states, causing relaxation, lysis, and release of insulin from konjac glucomannan (KGM).

While PBA, GOx, and ConA remain the most widely used glucose-responsive units in carrier-based systems, recent years have witnessed the emergence of an alternative paradigm: directly engineering insulin molecules to achieve glucose responsiveness. A landmark example is NNC2215, in which a glucose-binding macrocycle is conjugated to insulin, enabling glucose-dependent conformational changes that modulate insulin receptor activity (69). Although this molecular conjugation strategy falls outside the scope of material-driven systems reviewed here, it represents an important complementary approach that may inform future carrier design or eventually lead to carrier-free glucose-responsive insulin therapies.

## 3 Carrier materials

The selection of carriers is undoubtedly the most critical consideration in the design of smart insulin delivery systems. Carriers with distinct properties and functionalities—such as cationic polymers (70) and acid-degradable materials—often dictate the architecture of insulin delivery systems, enabling glucose-responsive insulin release through diverse mechanisms. These carrier materials can encapsulate insulin and glucose-sensing components to form hydrogels (66, 71–74), microneedle patches (48, 75–79), or nanoparticles (80–83). Below, we summarize several commonly used functional carriers and elaborate on their application in the rational design of glucose-responsive insulin delivery systems, supported by representative examples.

### 3.1 Metal-organic frameworks (MOFs)

MOFs represent a family of crystalline porous materials, distinguished by their intricate, periodic network structures. The use of MOFs as drug carriers has attracted significant attention and popularity in recent years (84–88). MOFs often exhibit sensitivity to various stimuli, such as pH, adenosine triphosphate, or glutathione, leading to their disintegration under specific conditions (89). Additionally, the adjustable inner cavity size and rigid structure of MOFs offer protection to encapsulated substances, shielding them from destructive inactivation. Enzymes, in particular, are highly sensitive to environmental temperature and pH, making the use of MOFs valuable for preserving enzyme activity. Likewise, MOFs have demonstrated efficacy in preserving the activity of protein-based drugs. However, challenges such as the long-term *in vivo* degradation behavior of MOFs, potential biotoxicity of certain metal ions, and complexities in large-scale production must be addressed before clinical translation.

**Figure 3:**
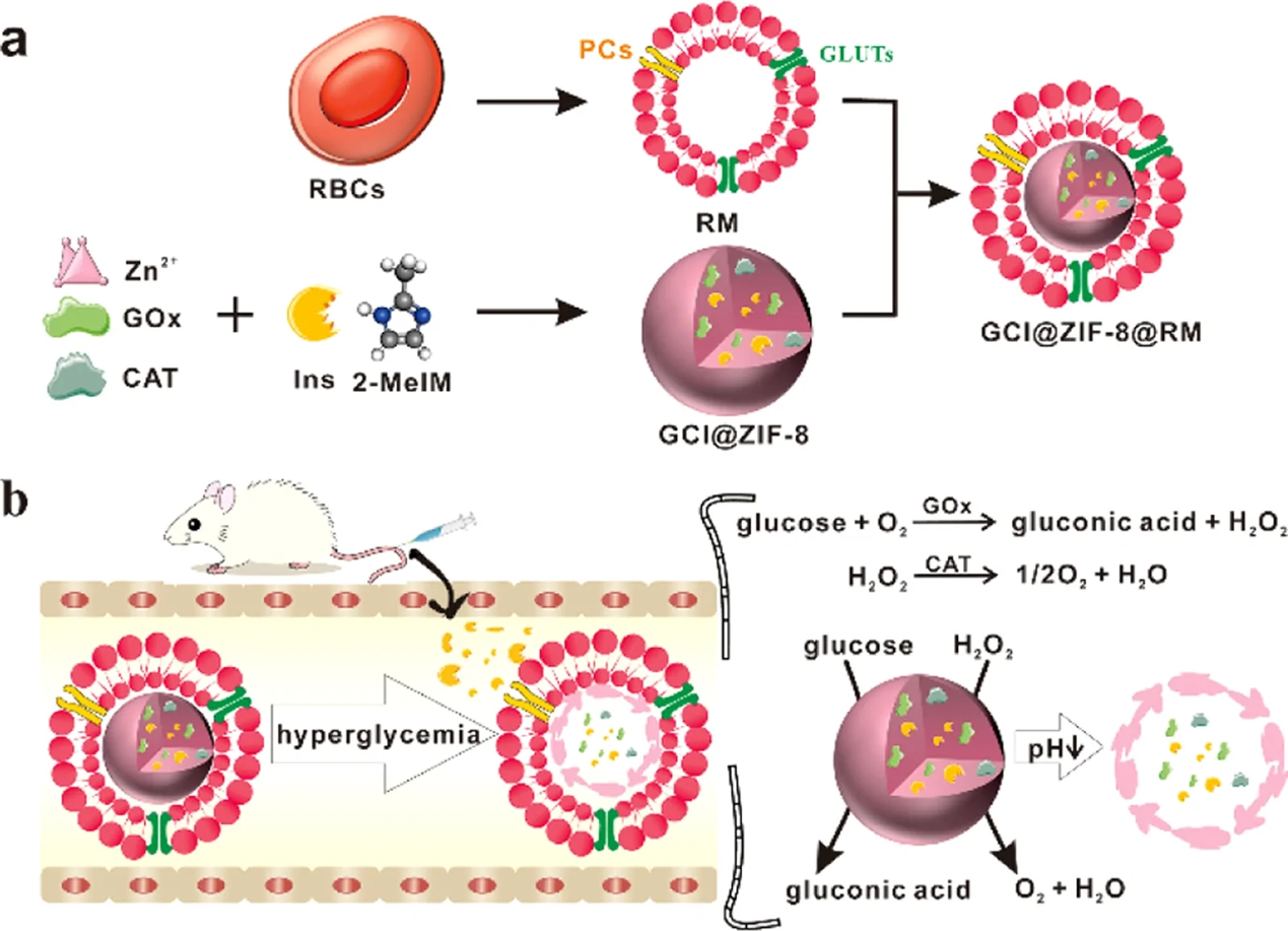
Biomimetic ZIF-8 nanoplatform for glucose responsive insulin delivery. **(a)** The biomimetic nanoplatform is constructed by coencapsulating insulin and an enzyme cascade within a ZIF-8 core enveloped by natural erythrocyte membranes. **(b)** Glucose responsive insulin release is achieved through the enzymatic oxidation of glucose into gluconic acid which triggers the pH dependent structural dissociation of the ZIF-8 framework to enable precise and on demand secretion. *In vivo* studies in diabetic mice demonstrate that this system effectively maintains glycemic homeostasis and ensures high biosafety through the synergistic elimination of oxidative byproducts (93). Copyright 2021, ACS. Reprinted with permission from American Chemical Society (License No. 6244640617495).

The integration of stimuli responsive disintegration with superior protective encapsulation is effectively demonstrated in the design of glucose responsive insulin delivery systems. In these advanced platforms, the pH sensitivity of certain MOFs is ingeniously leveraged to act as the core transduction mechanism, directly coupling physiological glucose levels to the on-demand release of insulin. The underlying principle relies on the enzymatic conversion of glucose by co-encapsulated GOx into gluconic acid, which induces a localized decrease in pH. MOFs engineered with pH-labile coordination bonds, such as the zeolitic imidazolate framework ZIF-8 (90), undergo controlled dissolution or structural rearrangement in response to this acidification, thereby releasing insulin in a glucose-concentration-dependent manner (91–93). Furthermore, to address the potential cytotoxicity of H_2_O_2_—a byproduct of the GOx reaction—strategies such as co-encapsulating catalase or incorporating catalytic metal ions (e.g., Co²^+^) have been employed. *In vivo* evaluations in diabetic mice demonstrate that this biomimetic nanoplatform effectively mimics the dynamic secretion patterns of pancreatic beta cells to achieve long term glycemic homeostasis with minimal immunogenicity and systemic toxicity (**Figure 3**). To achieve sustained release kinetics and improve biocompatibility, such MOF-based systems are often integrated into injectable hydrogel matrices. Alternatively, formulating these MOF composites into microneedle patches offers a minimally invasive and easily removable administration format (94). The design paradigm extends beyond ZIF-8. For instance, MOFs constructed from Zn²^+^ and 4,5-imidazoledicarboxylic acid (IDA) also exhibit pronounced pH-sensitive behavior. Researchers have leveraged this property to load GOx and the small-molecule drug deferoxamine mesylate (DFO) for diabetic wound healing (77). In this system, the glucose-triggered pH drop not only controls drug release but also activates the antioxidant function of DFO, which scavenges H_2_O_2_ generated during wound repair, demonstrating a sophisticated multifunctional therapeutic approach.

### 3.2 Polymers

In recent years, the most widely utilized carrier materials can be broadly categorized into cis-1,2/3-diol systems, cationic polymers (95), and pH-responsive materials. Among them, phenylboronic acid has a high affinity for compounds containing the cis-1,2/3-diol structure, and reversible boronate ester bonds can be formed. The combination of phenylboronate and GOx endows it with dual reactivity towards glucose and H_2_O_2_, allowing for the precise control of drug release according to physiological conditions. In addition, when glucose-sensitive substances such as GOx are conjugated with cationic polymers, the responsive discharge of negatively charged insulin can be achieved through the modulation of electrostatic interactions. Similar to pH-sensitive MOFs such as ZIF-8, certain polymers exhibit changes in their properties under acidic conditions, leading to degradation or depolymerization and subsequent release of insulin. These polymers can not only serve as carrier materials individually but also achieve a synergistic effect through cross-linking (96–101) which concurrently enhances the mechanical stability of the matrix and the sensitivity of the glucose triggered transition. This synergistic integration effectively prevents the premature leakage of insulin while accelerating the dissolution rate of the carrier upon reaching the glucose threshold. Additionally, they can be combined with glucose-sensitive elements to enable multiple responses to fluctuating blood glucose levels through concentration dependent secretion. Examples of studies of these materials over the past few years are presented in **Table 1**, and the subsequent subsections provide a comprehensive analysis of their specific performance and structural characteristics to offer a clearer understanding of the underlying glucose responsive mechanisms.

**Table 1:** List of the carrier materials mentioned above, their classification, stimulus response and target, and dosage forms of the drugs.

Classification	Materials	Response target	Stimulus response	Drug name	Dosage form	Disease	Refs.
Cis-1,2/3-diol systems	SA	PBA+ GOx	Glucose+H_2_O_2_	Insulin	Nanoparticle	Diabetes; Maintain normal BGL time:14h	(102)
	KGM	PBA	Glucose	Insulin+ liraglutide	Hydrogel	Diabetic nephropathy	(103)
		ConA	Glucose	Insulin	Nanoparticle	Diabetes	(68)
	PEG	GOx	pH+ H_2_O_2_	Curcumin	micell	cancer	(104)
	OS	ConA	Glucose	Exenatide	Nanogel	Diabetes; Maintain normal BGL time:48h	(105)
pH-sensitive materials	ZIF-8	GOx	pH+ Glucose	Insulin	Nanocrystal	Diabetes; Maintain normal BGL time:72h	(92)
	DEAEMA	GOx	pH+ Glucose	Insulin	Polymersome	Diabetes	(106)
	PSA	GOx	pH+ Glucose	insulin	Nanoparticle	Diabetes	(106–108)
	Ac-Dex SF MNs	GOx GOx	pH+ Glucose pH+ Glucose	Insulin insulin	Nanoparticle micro needle	Diabetes; Maintain normal BGL time:16h Diabetes	(108)
							(109)
Cationic polymers	PEI	GOx	pH+ Glucose	insulin	Electrostatic complexes	Diabetes; Maintain normal BGL time: at least 9h	(110)
	Silk fibroin with ϵ-PLL	GOx	pH+ Glucose	insulin	Hydrogel	Diabetes	(65)
	Polyacrylamide	PBA	Glucose	glucagon	Microneedle patch	Hypoglycemia	(111, 112)
	Polymers with Lewis base	PBA	Glucose	insulin	Microneedle patch	Diabetes	(77, 113, 114)

#### 3.2.1 Cis-1,2/3-diol systems

The discovery that PBA and its derivatives can selectively bind to molecules with specific diol structures has led to a surge of interest in their use for glucose-responsive drug delivery systems. In these systems, a polymer with a cis-diol structure serves as the delivery substrate and the concentration of glucose determines the rate of insulin release.

Traditionally, the materials used for insulin delivery have relied on a dynamic covalent cross-linked glucose-sensing system based on the interaction between PBA and diols. In this system, competition from ambient glucose reduces the cross-linking density of the material, leading to accelerated release of encapsulated insulin. Recently, researchers have focused on improving drug loading capacity and minimizing insulin leakage (115).

**Figure 4:**
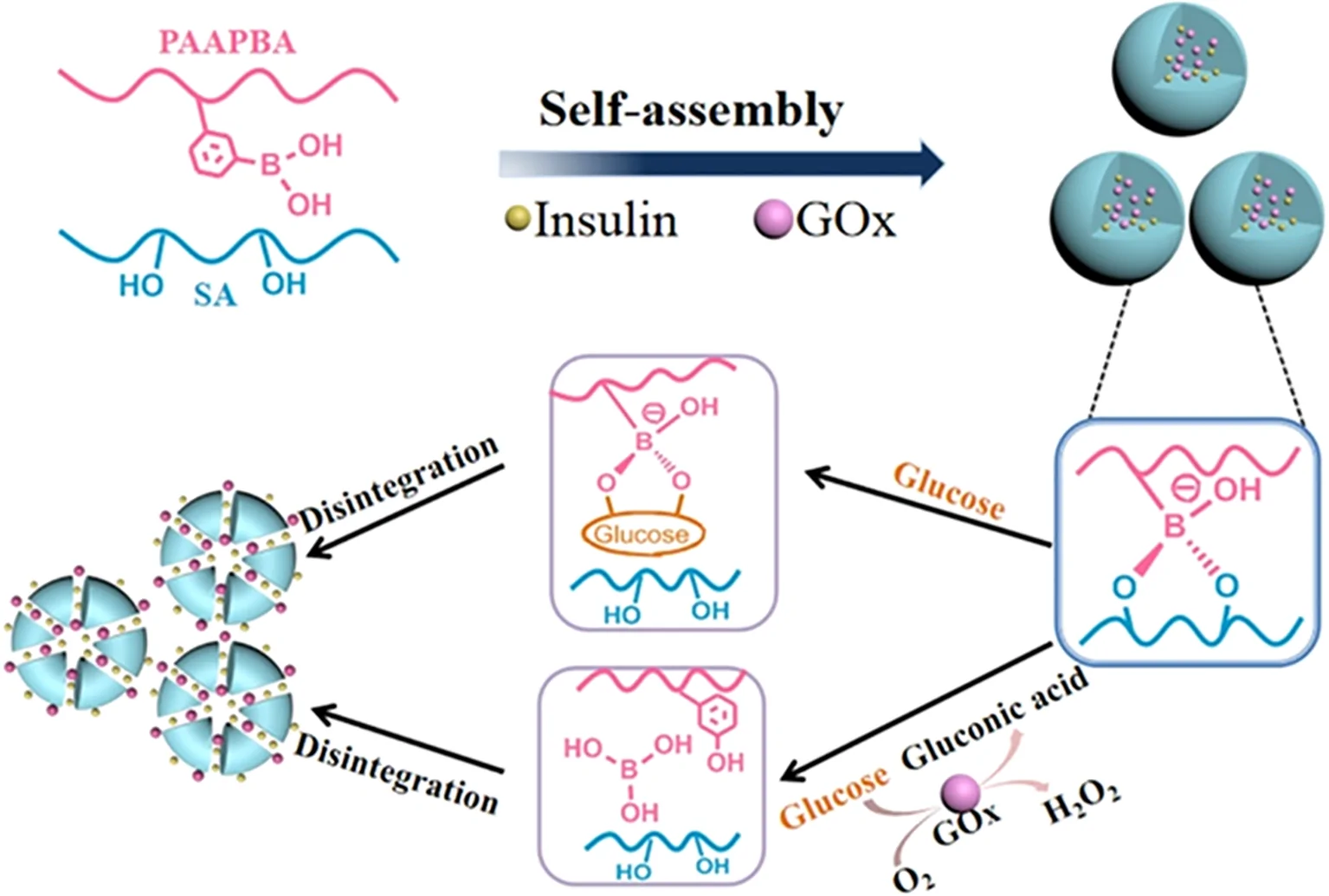
Fabrication of insulin/GOx-loaded nanoparticles and the glucose and H_2_O_2_ dual-responsiveness mechanism of cyclic boronic esters (102). Copyright 2020, Elsevier. Reprinted with permission from Elsevier (License No. 6045921490007).

Z. Chai and co-workers developed a polyacrylamide phenylboronic acid that can cross-link with sodium alginate (SA), which possesses a diol structure, to form a polymer framework via a boronic ester bond (102). The cyclic boronic ester serves as a functional group with dual responsiveness to glucose and hydrogen peroxide. As shown in **Figure 4**, glucose exhibited a strong affinity for polyacrylamide phenylboronic acid, displacing the cis-dihydroxy group on SA, which was originally bound to the phenylboronic acid in the polymer. This depolymerizes the carrier polymer, thereby releasing the encapsulated insulin. Second, the encapsulated GOx enzyme converts glucose to gluconic acid and H_2_O_2_. H_2_O_2_, in turn, degrades the boronic acid ester bond, facilitating the binding of glucose to phenylboronic acid. This accelerates depolymerization and promotes the release of insulin. The nanoparticle-based system, which encapsulates insulin and GOx, combines the PBA and GOx systems, resulting in enhanced effectiveness in the blood glucose response. In mouse studies, this system maintained normal blood glucose levels for up to 14 hours, underscoring its potential for prolonged and responsive insulin delivery.

KGM is another cis-1, 2-diol compound. M. Q. Tong et al. prepared KGM/PBA-PGA hydrogels as delivery carriers for insulin and peptides using a dynamic boron ester bond between phenylboronic acid grafted γ -polyglutamic acid and KGM (103). Typical cis-diol structures include polyethylene glycol (PEG). PEG is an oligomer or a polymer of ethylene glycol or ethylene oxide. It exhibits excellent water solubility, making it compatible with a wide range of organic compounds. PEG is nontoxic, nonirritating, highly stable under general conditions, and highly biocompatible. Due to its lack of spatial site resistance, PEG is frequently used in pharmaceutical formulations (24, 116). With a structure similar to that of cis-diol, PEG is often grafted with PBA and employed as a carrier for insulin (117). When GOx is introduced into the system, the polymeric carrier can exhibit both H_2_O_2_-responsive and pH-responsive behaviors because GOx can oxidize glucose to gluconic acid and H_2_O_2_. Thus, this multifunctional material has attracted considerable research attention.

S. Dong et al. synthesized an oxidative and pH dual-responsive cationic block copolymer, PEG-b-P (METMA), through several steps using methionine- (2-methacryloylethyl) ester and PEG (104). This copolymer can form polyionic complex micelles with GOx at pH 5.8 through electrostatic interactions. GOx-loaded nanogels composed of this copolymer exhibited glucose-responsive drug release. Similarly, X. Liu and colleagues have crafted a polymeric micelle system, ingeniously self-assembled from PEG-b-PAPBE, a construct where the hydrophilic PEG segment forms the outer shell, while the hydrophobic PAPBE component endows the micelle with a dual sensitivity to glucose and hydrogen peroxide (H_2_O_2_) (118). The micelle’s embedded phenylboronic esters are uniquely positioned to engage with glucose through substitution and to undergo hydrolysis in the presence of H_2_O_2_, culminating in the disintegration of the polymeric assembly. The strategic co-encapsulation of GOx within these micelles amplifies the system’s glucose-responsiveness, enhancing the release of insulin in a manner that is both sensitive and controlled. Furthermore, the glucose-catalyzed oxidation process generates H_2_O_2_, which in turn catalyzes the hydrolysis of phenyl borate, thereby integrating an additional layer of responsiveness into the system.

These examples highlight the use of compounds with cis-diol structures such as SA, KGM, and PEG in the development of glucose-responsive drug delivery systems. The incorporation of phenylboronic esters and GOx enables dual responsiveness to glucose and H_2_O_2_, offering precise control over drug release in response to physiological conditions.

#### 3.2.2 Cationic polymers

Insulin, with an isoelectric point of 5.1~5.3, carries a negative charge under physiological pH conditions. Researchers have leveraged this property to successfully encapsulate insulin using simple cationic polymers, such as polyethyleneimine (PEI) (110) and PEG-b-P (METMA) copolymer (104). In conjunction with GOx, insulin undergoes a charge conversion from negative to positive, allowing its release from the polycation when pH decreases in a hyperglycemic environment. This straightforward delivery approach often results in high drug loading and exhibits an effective glycemic response. A similar approach involving a cationic polymer matrix and GOx was employed by Tao et al. (65), where serine protein was chemically modified with poly- (L-lysine) (PLL) to create a cationic serine protein hydrogel system, which is based on the same principle as the polyethyleneimine described above. Upon addition of GOx, the hydrogel matrix swelled in a high-glucose environment, resulting in the release of the loaded drug.

In the absence of GOx to facilitate a decrease in pH, the scientific community (2, 52, 78, 79, 112, 119, 120) has made strides in investigating cationic polymers derived from 4-carboxy-3-fluorophenyboronic acid (FPBA), which are conjugated with the biodegradable PLL. The synthesis of these cationic polymers has been meticulously crafted to encapsulate the negatively charged insulin molecules effectively. Upon contact with glucose, the FPBA moiety undergoes a binding event that prompts a shift in its apparent pKa, imparting a negative charge upon the FPBA segment within the polymer. This charge alteration diminishes the pKa of the polymer, leading to a reduction in the overall positive charge density. Consequently, the electrostatic affinity between the polymer and insulin is attenuated, which in turn, initiates the insulin’s disengagement from the polymeric complex. Wang group has excelled in the modification of the biodegradable polymer PLL, integrating PEG and FPBA to synthesize a novel glucose-responsive polymer, designated as PEG-PLL-FPBA (121–123). Under conditions of low glucose concentration, this ingenious polymer adeptly captures negatively charged insulin, culminating in the formation of nano-insulin complexes (NICs) with exceptional insulin loading efficiency. Conversely, in an environment rich in glucose, the glucose-induced binding to the FPBA segment results in a significant diminishment of the polymer’s positive charge density. This charge alteration facilitates the strategic release of insulin, demonstrating the polymer’s sensitivity to glucose levels and its potential role in the precise regulation of insulin delivery.

Importantly, this PEG-PLL-FPBA-based system has been validated in large animal models. Zhang et al. demonstrated that a single subcutaneous injection of this glucose-responsive insulin complex maintained normoglycemia for up to one week in diabetic minipigs without inducing hypoglycemia or notable fibrous capsule formation (2). Furthermore, Liu et al. developed a long-acting glucose-responsive insulin formulation (GRIF) from glucosamine-modified insulin aspart and PLL-FPBA, which exhibited swift onset of action and superior blood glucose control in both type 1 diabetic mouse and minipig models (122). These findings in minipigs, whose metabolic and physiological profiles more closely resemble those of humans than rodents do, provide critical translational validation for this cationic polymer-based approach.

**Figure 5:**
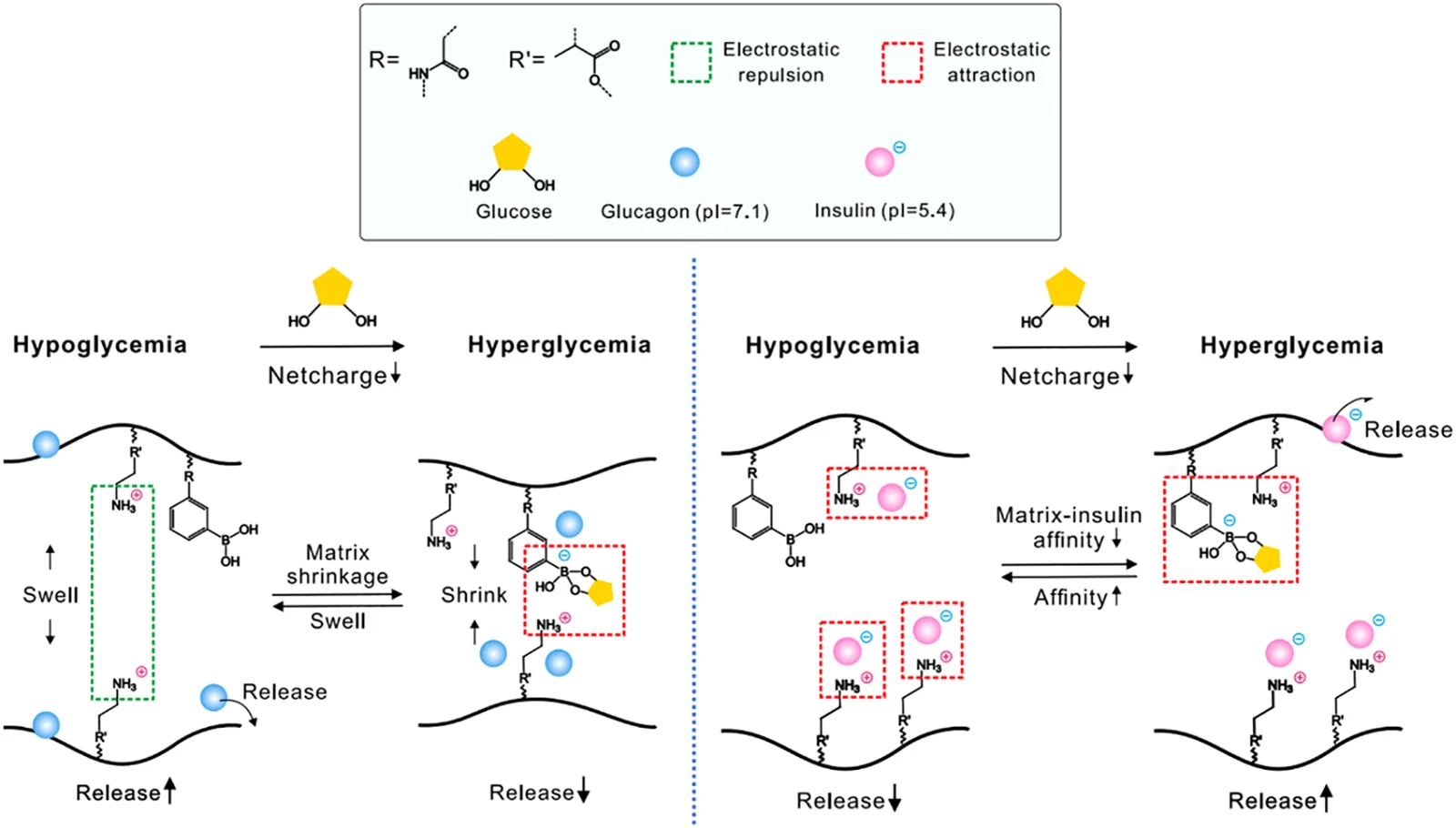
Mechanisms underlying glucose-regulated insulin/glucagon release (114). In the state of hyperglycemia, glucose will form a negatively charged complex with borate. The repulsion between positively charged polymers is weakened, which leads to polymer contraction and slows down the release of glucagon. And the electrostatic attraction between insulin and the complex will also be weakened, thus releasing insulin. Copyright 2020, the PNAS license. Creative Commons Attribution 4.0 International (CC BY 4.0).

Inspiration can be achieved by the formation of negatively charged complexes upon binding of phenylboronic acid to glucose. Z. Gu et al. synthesized polymers of (2-acrylamidoethyl) carbamate (EDAA) and 3-fluorophenylboronic acid (76). These cationic polymers bind to insulin, which can be stabilized *in vivo* by the negative charge of insulin under physiological conditions. In a hyperglycemic state, glucose-reactive PBA binds to glucose, converting the charge of the polymer portion from positive to negative and releasing insulin from the complex. The same concept can be applied to glucagon delivery. **Figure 5** shows a schematic of the release of insulin and glucagon from the cationic matrix. Based on this concept, Gu group chose a cationic polyacrylamide matrix to prepare glucagon-loaded microneedles (111). In a hypoglycemic environment, the cationic matrix expands, forming a cavity owing to charge repulsion, thereby releasing glucagon. As the blood glucose concentration increases, phenylboronic acid binds to glucose, converting it to a negative charge, neutralizing the surrounding repulsion, and inducing polymer contraction to slow down glucagon diffusion. Notably, this microneedle platform has been extended to glucose-responsive insulin delivery and validated in large animals. Yu et al. evaluated a glucose-responsive insulin patch in diabetic minipigs, showing that a single removable transdermal patch could effectively regulate blood glucose levels without causing skin irritation or systemic toxicity (113). Similarly, Yang et al. developed a dual-hormone (insulin and glucagon) microneedle patch and demonstrated closed-loop glucose control in both mice and pigs (124). These large-animal studies underscore the translational potential of microneedle-based systems, while also highlighting challenges such as patch adhesion and consistent insertion depth that must be addressed prior to clinical use.

Beyond injectable and transdermal systems, oral glucose-responsive insulin delivery has also been explored in large animals. Ji et al. developed an orally administered glucose-responsive polymeric complex that achieved high-efficiency insulin delivery in both mice and pigs (112). In diabetic pigs, oral administration of this complex resulted in significant blood glucose reduction with minimal hypoglycemic risk, providing proof-of-concept for non-invasive glucose-responsive insulin therapy in large animals.

In addition, Lewis base can also act as a cation (77, 113, 114). Co-loading of a Lewis base with insulin in a microneedle matrix enabled the formation of a stable drug-carrying system. When PBA binds to glucose, generating a negative charge, the increased negative charge enhances internal electrostatic repulsion, causing the microneedle matrix to expand and rapidly release insulin to regulate blood glucose levels. Conversely, when blood glucose levels decrease, the polymer matrix system returns to a stable state, with electrostatic attraction between positive and negative charges slowing the release of insulin.

Cationic polymer-type matrices will be well-developed for insulin delivery in the future. Choosing an appropriate cationic polymer matrix not only allows for high drug loading by utilizing electrostatic adsorption to stabilize encapsulated insulin and minimize leakage but also enables rapid glucose responsiveness. In the absence of a suitable cationic polymer, a certain amount of Lewis base can be added to the common hydrogel material function as a carrier, achieving the same glucose-responsive insulin release by exploiting the changes in matrix swelling sizes under different glucose conditions. This strategy offers high drug loading and rapid response. However, the cytotoxicity of traditional cationic polymers must be mitigated through degradable backbones or PEGylation. The incorporation of FPBA ingeniously combines the molecular sensing function of PBA with the drug-carrying function of the polymer.

#### 3.2.3 Acid-degradable polymers

pH-responsive materials are frequently employed in conjunction with the GOx (125, 126). D. Zhou and co-workers utilized the pH-sensitive monomer 2- (diethylamino)ethyl methacrylate (DEAEMA) to create polymeric membranes (106). When glucose enters the lumen and is oxidized by GOx to gluconic acid, ambient pH decreases. Subsequently, DEAEMA is partially protonated and becomes hydrophilic, resulting in significant swelling and enhanced permeability of the membrane, facilitating the release of internally encapsulated insulin. Moreover, polyamine salt aggregates (PSA) also possess pH-triggered properties and can be easily prepared, offering a high drug-loading capacity (107).

**Figure 6:**
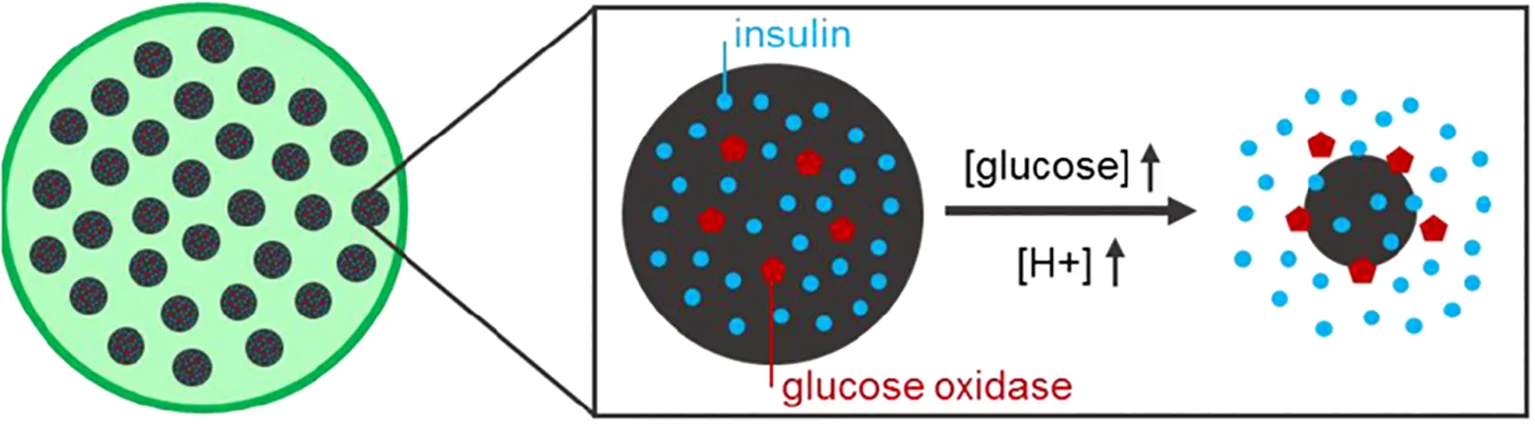
Schematic of encapsulation and glucose-responsive insulin release from acid-degradable Ac-dex NPs. The hierarchical assembly utilizes the larger size of the nanoparticles to prevent their premature leakage from the microgel pores while allowing the free diffusion of insulin upon nanoparticle degradation (66). Copyright 2021, Elsevier. Reprinted with permission from Elsevier (License No. 6045921334905).

Another polymer with acid-degradable characteristics is acetylated dextran (Ac-Dex), synthesized by the reaction of dextran with methoxypropene. Researchers (108) have further fine-tuned the amount of cyclic acetal to control the rate of insulin release and achieve both immediate and sustained drug release. The glucose responsive mechanism hinges on the enzymatic oxidation of glucose to gluconic acid which triggers the hydrolysis of the acid sensitive acetal groups within the acetylated dextran polymer matrix. This structural degradation leads to the dissolution of the nanoparticles and the subsequent release of encapsulated insulin at a rate that is precisely modulated by the local glucose concentration. L.R. Volpatti has ingeniously encapsulated glucose-responsive nanoparticles within the embrace of porous microgels (66). As shown in **Figure 6**, these nanoparticles, crafted from the pH-sensitive polymer Ac-Dex, are incorporated with insulin, GOx, and catalase. This formulation exhibits an agility in response to fluctuations in glucose concentration, reflecting the system’s high sensitivity. The microgels ensure the stability and longevity of the formulation, thereby facilitating a sustained and glucose-responsive insulin release. Experimental data from this study indicate that the system exhibits remarkable sensitivity, with the ability to restore normoglycemia in diabetic mouse models within two hours of administration. Furthermore, the formulation maintains stable blood glucose levels for up to six hours and demonstrates a significant reduction in hypoglycemic risks during glucose tolerance tests. These performance metrics highlight the agility of the system in response to fluctuations in glucose concentration and underscore the potential of microgel scaffolds in preserving the stability and longevity of responsive nanoparticles.

Building upon pH-responsive systems, the preparation of CMC-pHEA hydrogels has been achieved through the grafting of 2-hydroxyethyl acrylate (2-HEA) onto the backbone of carboxymethyl cellulose (CMC) (62). CMC-pHEA hydrogels have a higher swelling rate at elevated pH. Glucose levels can be measured using colorimetric or fluorescence imaging methods. The exploration of polymeric materials with acid degradability holds promise for the development of innovative insulin delivery strategies.

### 3.3 Bio-derived materials

Erythrocyte membranes, the naturally occurring lipid bilayer membranes of biological origin, hold significant potential for the delivery of exogenous molecules. They possess inherent biocompatibility and are often utilized as a protective “shell” on the surface of insulin-carrying particles to evade immune system clearance, thus enabling their use in intravenous injection. Additionally, red blood cells exhibit an extended lifespan and can remain stable in the circulation for prolonged periods, making them an ideal candidate for enhancing the pharmacokinetics of drug delivery systems (127). The greatest advantage of biomaterials is their superior biocompatibility. However, issues such as source variability, batch differences, and potential immunogenicity exist. Engineering modifications and standardized production are key focus areas.

M. He et al. designed a drug delivery system consisting of a ZIF-8 nanoparticle loaded with insulin, GOx, and catalase as the “core,” which was then enveloped by an erythrocyte membrane (93). This erythrocyte membrane enhanced the biocompatibility of the drug delivery platform and enabled intelligent intravenous insulin delivery. It effectively maintained normal blood glucose levels in diabetic mice for up to 24 hours. Similarly, Xu et al. developed glucose-responsive nanoparticles camouflaged with red blood cells to evade immune recognition, allowing these erythrocyte-coupled nanoparticles to circulate in the bloodstream for an extended duration, further enhancing their therapeutic efficacy (80).

**Figure 7:**
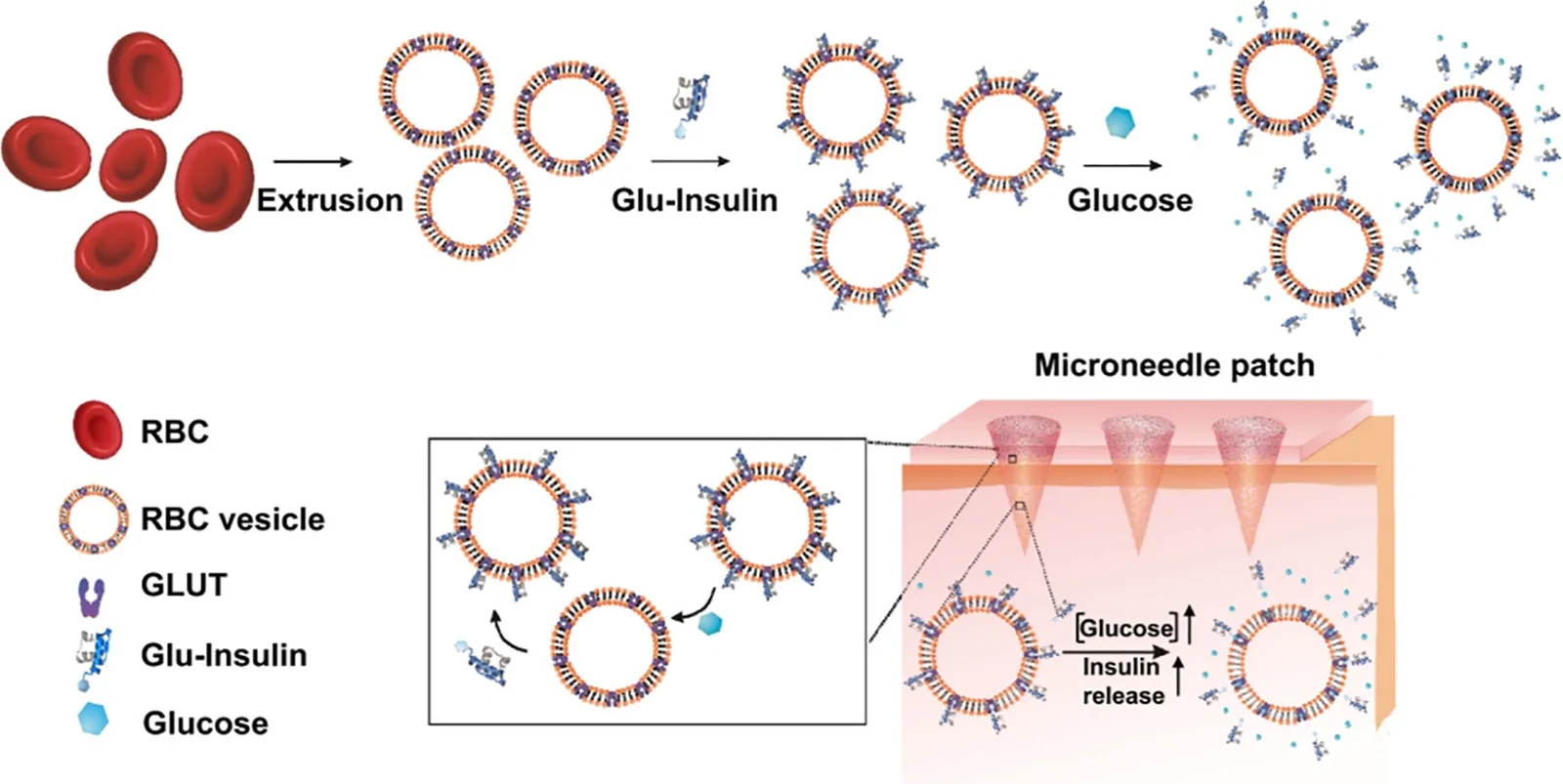
Glucosamine-modified insulin (Glu-Insulin) binds to RBC vesicles. Due to the competitive interaction between insulin and glucose, glucose-insulin can be released quickly under the condition of hyperglycemia (128). Copyright 2023, ACS. Reprinted with permission from American Chemical Society (License No. 6257091087091).

In 2022, Z. Gu ‘s team (128) developed a microneedle patch loaded with red blood cells. As depicted in **Figure 7**, red blood cells possess glucose transporters (GLUTs) on their surface, and insulin is modified with glucosamine, which binds to the GLUTs of red blood cells. When glucose concentration increases, glucose competes with insulin-carrying glucosamine for binding to the GLUT site, triggering the rapid release of insulin and thus regulating blood glucose levels. To enhance insulin delivery capacity, liposome nanoparticles containing GLUT were synthesized, mimicking the role of red blood cells and enabling the loading of additional “reserve” insulin to augment drug delivery capacity.

## 4 Conclusions and future perspectives

Recent advances in glucose-responsive insulin delivery have been propelled by the exploration of diverse sensing mechanisms and carrier systems. While traditional components such as PBA, Gox, and ConA have laid a substantial foundation, emerging strategies including those leveraging glucose transporters present novel pathways for active substrate translocation and controlled insulin release. Concurrently, a broad spectrum of carrier materials, including engineered metal-organic frameworks, pH-sensitive polymers, cationic polymers and bio-derived materials, has demonstrated significant potential for achieving controlled and long-acting therapeutic profiles.

Despite these advances, clinical translation remains elusive. To date, clinical investigation of glucose-responsive insulin analogs has been limited to the Phase 1 trial of MK-2640, an insulin-saccharide conjugate, which failed to demonstrate glucose-dependent changes in drug clearance, highlighting the challenge of achieving a robust ‘on/off’ activity ratio in humans (129).

The translational pathway for these systems may benefit from a holistic perspective that equally prioritizes biocompatibility, long-term stability, and safety alongside responsive performance. Encouragingly, several cationic polymer-based and microneedle-based systems have already demonstrated efficacy in minipigs and pigs (2, 76, 112, 113, 122, 124), providing critical translational validation beyond rodent models. However, long-term safety, immunogenicity, degradation profiles, and manufacturing scalability in large animals remain underexplored and warrant further investigation before clinical entry. Future material design could therefore integrate precise engineering strategies with the selection of benign components. Promising approaches include surface modification with zwitterionic or polyethylene glycol coatings to enhance stealth properties, as well as the incorporation of stimuli-degradable molecular linkers designed to ensure complete and safe metabolic clearance. In parallel, the ongoing refinement of sensing paradigms remains crucial. Although enzymatic systems like glucose oxidase provide high catalytic specificity, considerations regarding their long-term stability encourage the parallel development of robust non-enzymatic alternatives. The goal is to achieve an effective balance between catalytic efficiency and operational durability within the physiological environment.

Looking forward, the integration of multiple functions into a single platform represents a promising direction. Next-generation systems could be engineered to respond to complex diabetic microenvironmental cues, such as localized oxidative stress or specific enzyme activities, while also incorporating targeting motifs to improve spatial precision. This integrated approach has the potential to enhance therapeutic efficacy while minimizing off-target effects (130). Furthermore, the exploration of novel carrier matrices continues to offer new opportunities. For instance, nanoporous polymeric nanomembranes, with their tunable pore architecture and surface chemistry, present a versatile platform worthy of deeper investigation. Their adjustable permeability and high loading capacity are particularly relevant for the precise temporal control of insulin release, potentially serving as a key component in future integrated and multi-responsive delivery systems (131).

We envision that within the next 5–10 years, molecular-level glucose-responsive insulin conjugates (e.g., NNC2215) are the most likely to enter clinical trials due to their simpler regulatory pathway. Carrier-based systems such as cationic polymer complexes and microneedle patches may follow, pending further large-animal validation and manufacturing scale-up.

Ultimately, the progression of autonomous insulin delivery technologies will likely depend on the sustained convergence of intelligent material design with translational science. This encompasses the development of patient-centric formulations such as ultra-long-acting depots (132), alongside scalable manufacturing processes and rigorous pharmacoeconomic validation (133). Clinical evidence showing mixed glycemic benefits and risks of hybrid closed-loop systems further underscores the need for enhanced patient and provider education on this technology (134). By continuously uniting scientific innovation with practical clinical and engineering considerations, the field can move closer to creating viable, safe, and effective therapies that meaningfully improve diabetes management and patient quality of life.
