# Correction: Material-driven glucose responsiveness in insulin delivery systems: carrier selection and performance

**DOI:** 10.3389/fendo.2026.1974379

**Published:** 2026-09-16

**Authors:** Zhihan Li, Jingge Jiang, Ziyu Zhu, Cheng Luo, Chunyi Xu, Chuanpin Chen, Guoqin Ren, Ruofei Huang, Dongjuan He

**Affiliations:** 1 Department of Pharmacy, Xiangya School of Pharmaceutical Sciences, Central South University, Changsha, China; 2 The First Affiliated Hospital of Hunan Normal University/Hunan Provincial People’s Hospital, Changsha, China; 3 Quzhou People’s Hospital Affiliated of Wenzhou Medical University, Quzhou, China; 4 Zhejiang University, Zhejiang, China; 5 Hunan Institute of Drug Control, Changsha, China; 6 Yongkang First People’s Hospital of Wenzhou Medical University, Yongkang, China; 7 The Second People’s Hospital of Quzhou, Quzhou, China

**Keywords:** delivery systems, diabetes mellitus, glucose responsiveness, insulin, the carrier materials

Affiliation 7 “The Second People’s Hospital of Quzhou, Quzhou, China,” was omitted from the paper. This affiliation has now been added for author Dongjuan He.

The original version of this article has been updated.

